# The effect of antihypertensive agents on sleep apnea: protocol for a randomized controlled trial

**DOI:** 10.1186/1745-6215-15-1

**Published:** 2014-01-02

**Authors:** Fábio Tremea Cichelero, Denis Martinez, Sandra Costa Fuchs, Miguel Gus, Leila Beltrami Moreira, Flávio Danni Fuchs

**Affiliations:** 1 Postgraduate Studies Program in Cardiology, School of Medicine, Universidade Federal do Rio Grande do Sul, Porto Alegre, RS, Brazil; 2 Division of Cardiology, Hospital de Clinicas de Porto Alegre, Porto Alegre, RS, Brazil

**Keywords:** Sleep apnea, Hypertension, Treatment, Diuretics, Chlorthalidone, Amlodipine

## Abstract

**Background:**

Obstructive sleep apnea (OSA) and hypertension are well-known cardiovascular risk factors. Their control could reduce the burden of heart disease across populations. Several drugs are used to control hypertension, but the only consistently effective treatment of OSA is continuous positive airway pressure. The identification of a drug capable of improving OSA and hypertension simultaneously would provide a novel approach in the treatment of both diseases.

**Methods/Design:**

This is a randomized double-blind clinical trial, comparing the use of chlorthalidone with amiloride versus amlodipine as a first drug option in patients older than 40 years of age with stage I hypertension (140 to 159/90 to 99 mmHg) and moderate OSA (15 to 30 apneas/hour of sleep). The primary outcomes are the variation of the number of apneas per hour and blood pressure measured by ambulatory blood pressure monitoring. The secondary outcomes are adverse events, somnolence scale (Epworth), ventilatory parameters and C reactive protein levels. The follow-up will last 8 weeks. There will be 29 participants per group. The project has been approved by the ethics committee of our institution.

**Discussion:**

The role of fluid retention in OSA has been known for several decades. The use of diuretics are well established in treating hypertension but have never been appropriately tested for sleep apnea. As well as testing the efficacy of these drugs, this study will help to understand the mechanisms that link hypertension and sleep apnea and their treatment.

**Trial registration:**

ClinicalTrials.gov: NCT01896661

## Background

Obstructive sleep apnea (OSA) is a well-known cardiovascular risk factor and a major cause of secondary hypertension [1,2]. About 30% of the population suffers from OSA and it is moderate to severe (more than 15 apneas/hour of sleep) in 16.9% of adults [3]. OSA is observed in 30% to 80% of hypertensive patients [4]. We demonstrated that 38% of patients with controlled hypertension have OSA, in contrast to 71% of patients with resistant hypertension [5]. Each episode of apnea/hour increases the risk of hypertension by 4% [6].

The association between OSA and hypertension has been disregarded by clinicians and even by researchers of hypertension [7]. The high cost and low availability of the golden standard method for diagnosing OSA, full polysomnography, may be one of the reasons [8,9]. The use of portable devices, which have been validated in our laboratory, could circumvent this limitation, since they have reasonable sensitivity (96%) and specificity (64%) [10].

The standard treatment for OSA is continuous positive airway pressure (CPAP) and 46 clinical trials show the benefits [11]. It has also been shown in randomized clinical trials that CPAP lowers blood pressure, particularly in patients with hypertension [12]. Blood pressure decreased by 7.8/5.3 mmHg in the 24 h ambulatory blood pressure monitoring in patients with OSA and hypertension, but did not decrease in those without OSA [13]. The efficacy of CPAP in patients with milder forms of OSA is still unproven [14], which could be secondary to the low adherence in the use of the method. Other therapies could be beneficial in such patients [15].

A Cochrane review, which identified 26 clinical trials of 21 drugs totaling 394 patients, failed to identify any pharmacological treatment with consistent efficacy. Some drugs, like fluticasone, mirtazapine, physostigmine and nasal lubricants, seem to reduce the number of apneas, but the trials were small and had methodological limitations, precluding the use of these drugs in clinical practice [15]. It has been suggested that drug therapy must be tailored to the mechanism of OSA identified in each patient [15].

An extravascular fluid shift has been implicated in the physiopathology of OSA. During the night, a shift of fluids from the legs causes an increase in neck circumference, peripharyngeal pressure and upper airway collapsibility [16-19]. Almost 60 years ago an increase of 0.5 cm in the size of the earlobes during sleep was described [20]. The application of lower body pressure of 40 mmHg using antishock trousers reduces leg fluid volume and increases neck circumference and the resistance of the pharynx [18].

Patients with controlled hypertension underwent a reduction of 175 mL in leg volume and an increase of 1.0 cm in neck circumference after sleeping, in comparison with a leg volume reduction of 346.7 mL and an increase in the neck circumference of 1.5 cm in patients with resistant hypertension [21]. The leg volume shift is positively correlated to the number of apneas (*R*^2^ = 0.56) [21]. CPAP reduced the increase in neck size proportional to the reduction in the number of apneas, but it did not prevent the leg volume change [19].

Sympathetic renal ablation with radio-frequency waves reduced blood pressure by 33/11 mmHg in 6 months [22]. It also reduced the number of apneas/hour from 16.3 to 4.5 for ten patients with resistant hypertension and OSA [23], an effect that was attributed to the promotion of salt excretion and total body fluid reduction [23]. Spironolactone led to a reduction from 39.8 to 22.0 apneas/hour after 8 weeks of treatment of 12 patients with resistant hypertension [24]. There is no controlled study exploring the concept that these drugs may act through total body fluid reduction.

In the ALLHAT trial, chlorthalidone, lisinopril and amlodipine had comparable efficacy in the prevention of coronary heart disease [25]. The diuretic, however, was superior to lisinopril in the prevention of strokes and amlodipine in the prevention of heart failure [25]. There is evidence that the efficacy in the prevention of events is related to the magnitude of the blood pressure reduction [25,26].

The main adverse event for chlorthalidone is hypokalemia, which blunted the efficacy of the treatment in the SHEP trial [27] and increased serum glucose levels [28]. The use of amiloride, a physiological aldosterone antagonist, could ameliorate this adverse effect. This potassium-sparing diuretic was effective and well tolerated in a randomized trial performed by our group [29].

Amlodipine was more effective than valsartan, an angiotensin receptor blocker, in the prevention of myocardial infarction and stroke in the VALUE trial [30]. In the ACCOMPLISH trial, the combination benazepril-amlodipine was more effective in the prevention of composite cardiovascular events than the combination benazepril-hydrochlorothiazide [31]. There is no evidence that amlodipine influences the balance of fluids, and edema is one of its main adverse effects.

Overall the evidence shows that chlorthalidone and amlodipine are the most effective drugs for the initial treatment of hypertension. Their use in OSA has not been appropriately tested to date. Thus, a trial testing the efficacy of these drugs to control both blood pressure and sleep apnea is warranted. Such a trial could contribute to our understanding of the relation between hypertension, fluid levels, hypoxia and OSA.

### Rationale

OSA has been associated with fluid retention, which accumulates in the pharynx facilitating its collapse, generating intermittent hypoxia and increasing sympathetic activity and blood pressure. CPAP alleviates apnea, which reduces sympathetic activity, reducing blood pressure and increasing salt and water excretion. Sympathetic renal ablation promotes salt and water excretion, reducing systemic sympathetic activity and total body water (including the pharynx), thus alleviating apnea. Diuretics could be a new way to abort this vicious cycle by promoting the direct excretion of salt and water.

### Research question

Is chlorthalidone with amiloride effective in the treatment of OSA in comparison to amlodipine in patients with OSA and hypertension?

## Methods/design

This is a randomized double-blind clinical trial, controlled by an active treatment.

### Eligible participants

Eligible participants are patients older than 40 years of age with stage I hypertension (140 to 159/90 to 99 mmHg) and moderate OSA (15 to 30 apneas/hour of sleep).

### Exclusion criteria

Patients are excluded if they have a low life expectancy, other indications for the use of diuretics or calcium channel blockers, intolerance or contraindications to the study drugs, cardiovascular disease (heart failure or recent – within three months – myocardial infarction or stroke), secondary hypertension or participated in another clinical trial in the previous 6 months or if they are pregnant or use more than one drug for hypertension.

### Random allocation

Randomization will be done using a list generated by validated software (a random allocator), with a block size of four.

### Interventions

The interventions are chlorthalidone plus amiloride 25 mg and 5 mg daily, respectively, versus amlodipine 10 mg daily, taken in the morning.

### Outcomes

#### Primary outcomes

1. Number of apneas/hour (apnea-hypopnea index)

2. Blood pressure

#### Secondary outcomes

1. Adverse events

2. Somnolence scale (Epworth)

3. Respiratory parameters

4. C reactive protein

#### Follow-up and duration of the study

**Figure 1:**
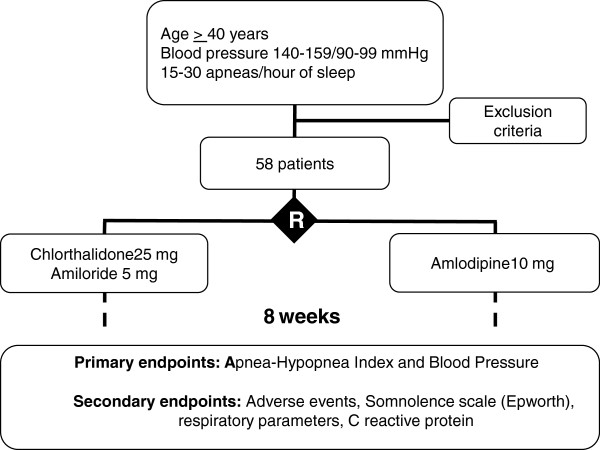
**Flow chart of selection**, **interventions and outcomes.**

There will be outpatient clinical visits for evaluation at enrollment and week 8 of treatment. Figure 1 is a flow chart for the selection, interventions, follow-up and outcomes.

#### Assessment of outcomes

The number of apneas/hour will be measured at the baseline and follow-up by type III portable polysomnography (Somnocheck, Weinmann GmbH, Hamburg, Germany), which was validated by us [10]. Average blood pressure (two measurements using a validated automatic electronic device) and ambulatory blood pressure (Spacelabs 90207, Spacelabs, Redmond, WA) will be measured at the baseline and follow-up.

Sleepiness will be measured using the Epworth somnolence scale at the baseline and follow-up. It records the likelihood that someone will fall sleep during eight daily activities (sitting and reading, watching television, sitting in a public space, being passenger in a car for 1 hour, lying down in the afternoon, sitting and talking to someone, sitting after a meal without alcohol and stopped in a car for few minutes).

Adverse events will be investigated using open questions and a semi-structured questionnaire, with questions on general symptoms and the presumed adverse effects of the drugs used in the trial. Standard laboratory tests will be used to identify adverse events, such as hypokalemia and elevated glucose levels. C-reactive protein levels will be determined as well.

### Wash-out

Patients taking an antihypertensive drug will need to stop it for 2 weeks prior to the study to be confirmed for eligibility, to allow time for most of the effects of the blood pressure drug to vanish [32].

### Control of adherence

Adherence will be check by counting pills.

### Sample size calculation

For a mean of 20 apneas/hour at the baseline and a reduction of 7 apneas/hour, with a standard deviation of 9 apneas/hour, power of 80% and *P* alpha of 5%, 26 patients will be required per group. The sample will be increased by 10% to account for possible losses in follow-up, so that 58 patients need to be randomized.

### Statistics

Differences between variables for the groups will be analyzed with chi-squared tests for categorical and Student’s *t* tests for continuous variables. Confounding will be controlled with logistic regression and multiple linear regression models.

### Ethical approval

The project and the informed consent form were approved by the ethics committee of the Hospital de Clínicas de Porto Alegre, which is accredited by the Office of Human Research Protections as an Institutional Review Board. All participants will be asked to sign the informed consent form prior to participation in the study.

## Trial status

The trial is currently recruiting patients.

## Abbreviations

CPAP: Continuous positive airway pressure; OSA: Obstructive sleep apnea.

## Competing interests

The authors declare that they have no competing interests.

## Authors' contributions

FTC conceived the study, revised the background, prepared the data collection plan and prepared the draft of the manuscript. DM participated in the revision of the background, participated in preparing the data collection plan and contributed to drafting the manuscript. SCF participated in preparing the data collection plan and contributed to drafting the manuscript. MG participated in the revision of the background, and revised the draft of the manuscript. LBM participated in preparing the data collection plan and revised the draft of the manuscript. FDF conceived the study, participated in preparing the data collection plan and prepared the final version of the manuscript. All authors read and approved the final version of the manuscript.
